# Management of Warfarin Anticoagulated Patients Choosing Hyperbaric Oxygen Treatments

**DOI:** 10.1055/s-0038-1676984

**Published:** 2019-01-07

**Authors:** Christina E. DeRemer, Stacey Curtis, Jason Konopack

**Affiliations:** 1 Department of Pharmacotherapy and Translational Research, University of Florida College of Pharmacy, Gainesville, Florida, United States; 2 Department of Clinical Practice, University of Florida College of Medicine, Gainesville, Florida, United States

## Clinical Question

Should anticoagulation management be adjusted in patients undergoing hyperbaric oxygen treatments?

## Evidence-Based Answer

Despite its effectiveness having unclear outcomes, hyperbaric oxygen therapy (HBOT) may be instituted as part of a treatment plan for ischemic stroke sequela, wound care, or numerous other medical conditions. 1 2 Due to additional comorbidities, these same patients may require anticoagulation management. Current literature lacks evidence of pharmacokinetic or pharmacodynamics changes in anticoagulation medication, specifically warfarin, used during HBOT. 3 Remaining is the question, does the increase in oxygen delivery at a higher than atmospheric pressure increase bleeding complications that would require adjustment to anticoagulation medication management? Resources indicate the common areas that may result in bleeding are epistaxis and tympanic membrane rupture that may have a connection to sinus cavity complications as an air cavity. 2 4 However, exploration of these associated bleeds is limited in the literature and this condition seems minor in nature, but severity could depend on patient characteristics such as blood pressure and diabetes. 2 Less common, but more significant in severity include pulmonary complications, central nervous system oxygen toxicity, and ocular complications, but occurrences are again limited in the literature. 2

## Case Report

S.B. is a 78-year-old Caucasian female who was prescribed rivaroxaban for atrial fibrillation, but stopped treatment due to risk concerns raised on television advertisements. Several months following her cessation of rivaroxaban therapy, she suffered an ischemic cerebral vascular accident (CVA) which converted to a hemorrhagic stroke 2 days later. These events, which occurred 2 years ago, resulted in left hemiplegia. While it is unclear from her medical report when warfarin was started, she has been taking warfarin for her atrial fibrillation and monitored at her local anticoagulation clinic for approximately 1 year.

Over that year of warfarin therapy, S.B. reported regularly to the anticoagulation clinic for international normalized ratio (INR) monitoring, generally with 5- to 8-week follow-up intervals. Most of the time, she remained within her prescribed therapeutic range (INR goal of 2–3). As she suffered from numerous sequelae related to CVA, she decided to pursue HBOT as a means to improve her clinical status and quality of life. The week prior to her HBOT, she arrived at the anticoagulation clinic for her regular follow-up, and it was determined that her INR was elevated at 3.5. During the ensuing discussion, her son, who was also her caregiver, mentioned that she would be undergoing HBOT for 1-hour sessions, five times a week, for 6 weeks of total duration of treatment. Due to the extended course of treatment and the fact that they would be devoting their resources to travel the long distance to the HBOT treatment site, the patient and her son were resistant to alter the warfarin dose at that time. Concerns for increased risk of bleeding events were discussed and the patient was scheduled for return monitoring. Her next INR continued slightly out of range at 3.2, and no dose change was made. At that time, she was 3 weeks into her HBOT treatment plan without any bleeding complications noted. Her INR 4 days after her last HBOT was within target range at 2.7, and again it was recorded that there had been no complication of bleeding during the treatments and no adjustment to warfarin dose.

## Practical Approach

Hyperbaric oxygen therapy has a limited number of accepted treatments in the United States compared with international practice; however, use from athletes has made HBOT a more acceptable treatment modality despite the lack of clinical trials and outcome data. 5 Regardless of the indication, there is limited literature outlining the affects and safety that this treatment modality has on drug response. Further complicating is that very likely providers and pharmacists lack the awareness that patients are undergoing HBOT and therefore are unable to provide impactful guidance for medication therapy management. Open discussion regarding nontraditional treatments may lead to more awareness, allowing providers the opportunity to assess patients at risk for potential complications. Additional research is required to define risk characteristics for a clearer safety profile as well as clinical efficacy in an array of therapy options. Currently, due to limited literature, routine management alteration may not be required for anticoagulation use during HBOT.
